# Antibiotic lock solutions as adjunct therapy for catheter-related blood stream infections in pediatric hemodialysis patients

**DOI:** 10.3389/fped.2024.1379895

**Published:** 2024-04-11

**Authors:** N. Blair, P. Patil, D. Nguyen, B. Paudyal-Nepal, F. Iorember

**Affiliations:** ^1^ School of Medicine, Texas A&M University School of Medicine, College Station, TX, United States; ^2^ Department of Pharmacy, Driscoll Children’s Hospital, Corpus Christi, TX, United States; ^3^ Department of Pharmacy, Children’s Healthcare of Atlanta, Atlanta, GA, United States; ^4^ Department of Nephrology, Texas Children’s Hospital, Austin, TX, United States; ^5^ Department of Nephrology, Driscoll Children's Hospital, Corpus Christi, TX, United States

**Keywords:** catheter related blood stream infections, antibiotic lock solutions, hemodialysis, antibiotic lock therapy, biofilm, hemodialysis catheter colonization

## Abstract

The predominant use of intravenous catheters as primary access type in the pediatric hemodialysis population is associated with an increased risk of catheter related blood stream infections. While strict adherence to catheter placement and long-term care guidelines have helped to decrease the incidence of these infections, blood stream infections remain an infection burden in pediatric patients with long term hemodialysis catheters. The formation of biofilms on the surfaces of these catheters has been shown to be a source of microbes causing blood stream infections. One of the strategies for preventing bacterial colonization, inhibiting microbial multiplication, and suppressing the seeding of these microbes from biofilms upon maturation, has been the use of antibiotic-based lock solutions in-between dialysis treatments. Although clinical guidelines for the use of antibiotic lock solutions are yet to be developed, available evidence suggests a beneficial role of antibiotic lock solutions in the management of catheter related blood stream infections. Additionally, a clear understanding of how biofilms are formed and their role in the pathogenesis of catheter related bloodstream infection will facilitate the development of solutions that can prevent biofilm formation and inhibit their multiplication, maturation and seeding into the bloodstream.

## Introduction

In the 2016 United States Renal Data System annual report (USRDS), 81.4% of incident pediatric kidney failure patients aged 0–21 years, needing renal replacement therapy, were initiated on hemodialysis through a central venous catheter (CVC) (1). This observation has been corroborated by the international pediatric hemodialysis network (IPHN) registry. Of the 404 pediatric patients entered into the registry from December 2012 through September 2017, 73% of them initiated hemodialysis through a CVC (2). Catheter related blood stream infections (CRBSI) are associated with CVC use and a significant cause of morbidity and mortality in hemodialysis patients (3–5). In its 2014 report, the National Healthcare Safety Network reported that most bloodstream infections (63.0%) and access-related bloodstream infections (69.8%) occurred in hemodialysis patients with a CVC (5). The cost of ambulatory care of CRBSI, and hospitalization of pediatric hemodialysis patients with CRBSI is substantial, ranging between $11,584–36,266 per patient, with higher costs in patients needing intensive care unit stay and CVC replacement (6, 7). Microbial colonization and biofilm formation on the surface of CVC occurs frequently in hemodialysis patients dialyzed through CVC, and has been identified as a source of the microorganisms causing CRBSI (8–11). Strategies to prevent CRBSI include sterile vascular access practices as recommended by the Centers for Disease Control (CDC), the use of sterile catheter locking solutions and more recently, anti-adhesive or bactericidal catheter surface modification (12–14). While arteriovenous fistulas and arteriovenous grafts have been shown to be associated with lower rates of access-related sepsis, improved patency rates, improved dialysis adequacy and overall lower morbidity and mortality compared to CVC, their placement is often hindered by age and small vessel size in the pediatric population (15, 16). In this narrative review article, we examine available evidence on biofilm characteristics and the benefits of antibiotic lock therapy (ALT) in the treatment and prevention of CRBSI in pediatric hemodialysis patients.

## Pathogenesis and treatment of CRBSI in pediatric hemodialysis

Multiple risk factors have been identified and implicated in the pathogenesis of blood stream infections in pediatric patients with indwelling CVC and hemodialysis catheters including non-sterile technics during catheter use, the presence of gastrointestinal disease and young age (5, 17). Gram positive organisms are the cause of catheter related blood stream infections in 25.8%–63% of cases, depending on the study, with *Staph. aureus* and *Staph. epidermidis* being the predominant organisms identified in these infections (2, 3, 14). In the largest international pediatric hemodialysis cohort, CRBSI occurred in 1.3/1,000 catheter-days, with 63% of pathogens identified as gram positive organisms, and 17% as culture negative (2). In the standardized care to improve outcomes in pediatric ESKD (SCOPE) collaborative study, gram positive organisms were identified in 49.7% of cases, and gram negative organisms in 27.5% (3). Although less common, fungal pathogens such as Candida albicans also cause these infections. The sources of these organisms include colonization at the catheter insertion site, contamination of the catheter hub and hematogenous seeding of organisms from catheter biofilms and other infected sites (18–21).

### The biofilm

**Figure 1:**
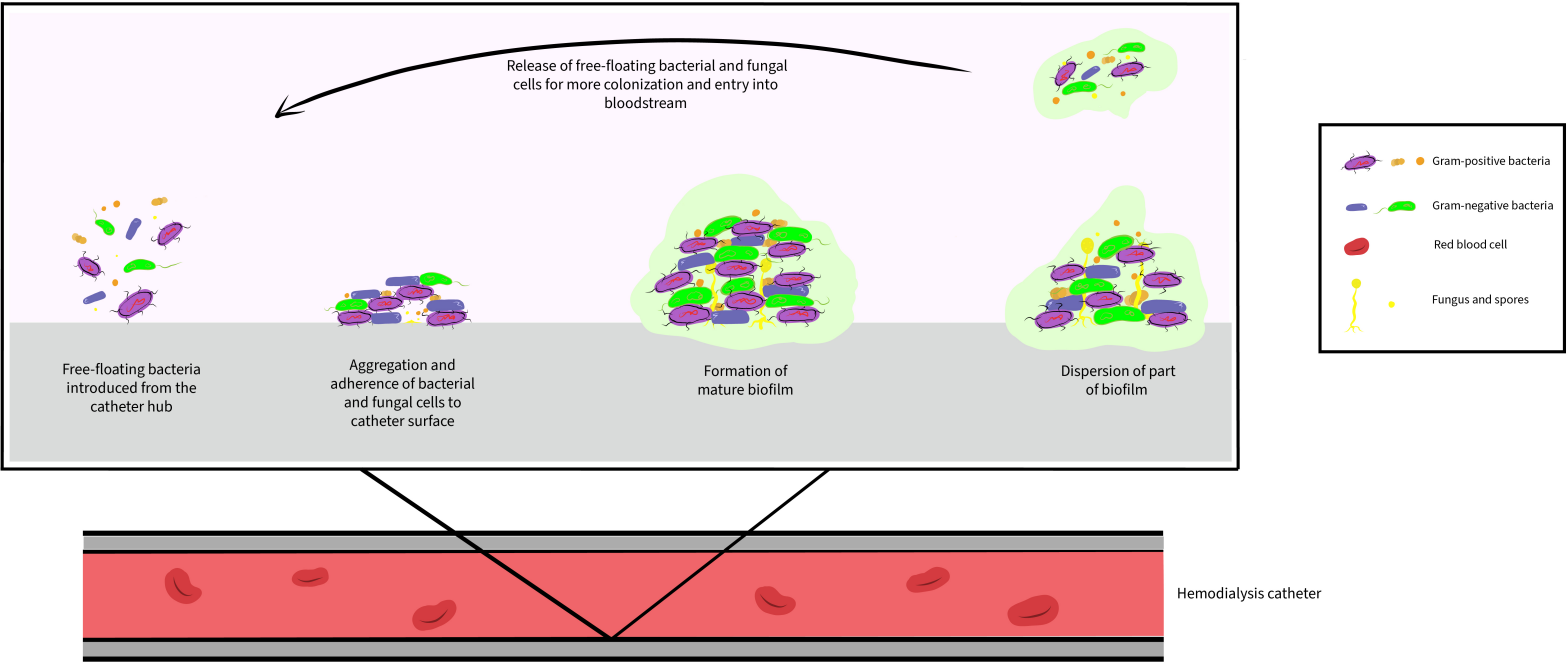
Biofilm formation on the intraluminal surface of hemodialysis catheter.

The biofilm is an organized and complex aggregate of microorganisms living within an extracellular polymeric matrix that they produce, from which bacterial seeding can occur (8, 9). The process of biofilm formation starts with the attachment of microorganisms to the surface of catheters, followed by multiplication of the microorganisms, maturation, and synthesis of a polymeric matrix. Upon maturation, the bacteria detach and disseminate into the blood stream, predisposing to systemic infection (Figure 1). In hemodialysis catheters, biofilms form on both extraluminal and intraluminal surfaces and are an important source of microorganisms in CRBSI (22–24). In an adult prospective observational study, Ramanathan and colleagues demonstrated the presence of bacterial growth on the inner and outer surfaces of cultured hemodialysis catheters in 62% bacteremic and 30% nonbacteremic patients. Bacteremic patients had thicker biofilms on all catheter surfaces, with the extraluminal segment outer surface biofilms being thicker, compared to the luminal surface (*p* < 0.001) (25). In another prospective study and using quantitative PCR (qPCR) targeting 16S rRNA, intraluminal bacterial colonization was detected in 60% of hemodialysis catheters (26). It is suggested that biofilm formation can occur in less than 3 days after catheter insertion (27) and promote the development of antibiotic resistance (28–30), leading to persistent or recurrent blood stream infections. Efforts to eradicate the biofilm have included the use of antibiotic lock solutions, first described by Messing et al. in 1988 in which the antibiotic lock technique was associated with shortening of the time to obtaining negative cultures and decreased length of hospital stay (31, 32).

### Approaches to treatment

Current CRBSI treatment approaches include the use of systemic antibiotic therapy alone or in combination with antimicrobial catheter lock solutions, removal and subsequent replacement of catheters or catheter exchange over a guidewire (33–35). The use of systemic antibiotics alone may be insufficient to eradicate bacteremia and has been shown to be associated with higher recurrence rate of CRBSI (34, 36). After appropriate blood cultures have been drawn, current recommendations are to initiate broad antibiotic coverage with Vancomycin and a third-generation cephalosporin or an aminoglycoside, while culture and sensitivity results are being awaited. Cefazolin may be used in place of Vancomycin in dialysis units with low prevalence of methicillin-resistant staphylococcus. When a catheter-related infection is documented and a specific pathogen is identified, systemic antimicrobial therapy should be narrowed based on sensitivity results and consideration given for antibiotic lock therapy, if the catheter is not removed. Recommended treatment duration is 3 weeks for uncomplicated bacteremia and 6 weeks for patients with metastatic infection such as endocarditis and osteomyelitis (34, 37).

## Antibiotic lock therapy

### Rationale and types

Strategies to reduce the risk of CRBSI in hemodialysis patients include decreasing the number of patients utilizing a hemodialysis catheter for chronic hemodialysis and the use of aseptic techniques during the connection and disconnection process of the hemodialysis procedure (38). More recently, the preemptive use of antibiotic lock therapy (ALT), a process of instilling high concentrations of an antimicrobial solution, typically along with an anticoagulant, to dwell in the catheter lumen when the CVC is not in use, has shown promise in helping decrease the rates of CRBSI (39–42). In a systematic review and meta-analysis of available randomized controlled trials that compared single or combination antimicrobial catheter lock solutions with non-antibiotic antimicrobial (antiseptic) solutions for the prevention of CRBSI in patients undergoing hemodialysis, antibiotic lock solutions were shown to significantly reduce the rate of CRBSI and catheter removal (43). In hemodialysis patients with established CRBSI, the adjunctive use of ALT was shown to be superior to systemic antibiotics alone in treatment of CRBSI (44). Data from Pediatric hemodialysis patients also shows that ALT is a viable option for attempting catheter salvage in CRBSI (45). The challenges with effective treatment of CRBSI in hemodialysis patients likely reflects the issues with achieving therapeutic antibiotic concentrations to successfully eliminate microbial biofilms on the surfaces of catheters (41, 46). Biofilms exhibit mechanisms which promote the survival of bacteria within the extracellular matrix including antibiotic efflux, reduced permeability to antibiotics, activities of enzymes that modify or destroy antibiotics, and modification of the antibiotic target through mutation, enzymatic action, or the presence of an alternate target (47). It is now understood that the bacteria within biofilms can withstand high concentrations of antibiotics and resist killing even when they are susceptible to the antimicrobial agents *in vitro*, making bacterial eradication challenging in clinical settings, and putting the patient at risk for recurrent CRBSI. High concentrations of antimicrobial solutions are an effort to address biofilm resistance and tolerance (47, 48). To improve the penetration and successful killing of biofilm bacteria, antibiotics must be at 100–1000 times the typical concentration (49, 50). Attempting to salvage an infected catheter with systemic therapy alone may not be enough and may increase the risk of complications such as endocarditis and epidural abscess, thus, the recommendation to fill catheter lumens with supratherapeutic antimicrobial concentrations and allow to dwell for hours to days, also known as antibiotic locks (51–56). Antibiotics are often mixed with heparin to help maintain lumen patency while the solution dwells. The mixture compatibility and stability are important factors in determining efficacy of antibiotic lock solutions (37, 45, 52, 57–60, 61, 62, 63). The ultimate choice of ALT depends on the microorganisms recovered on blood culture and their sensitivies to antibiotics. Commonly used antibiotic solutions and their characteristics can be found in Table 1.

**Table 1:** Antibiotic lock solution characteristics.

Antibiotic	Antibiotic concentration	Additive & concentration	Solution compatibility	Solution stability	References
Amikacin	2 mg/ml	Heparin 20 units/ml	Visual stability at 7 days at 25°C and 37°C in glass tubes confirmed	Dwell time minimum of 72 h; duration of 3–14 days	(57, 58)
Amphotericin B Liposome	2.67 mg/ml	Heparin 66.7 units/ml	Compatible for 72 h	24 h, refrigerated. Stable for 72 h	(58, 59)
Ampicillin	10 mg/ml	Heparin 10 and 5,000 units/ml	Compatibility established up to 24 h at room temperature	48 h, refrigerated. Stable for 14 days, yellow discoloration at 3 days	(37, 61)
Caspofungin	3.33 mg/ml	Heparin 200 units/ml	Compatible for 48 h	48 h refrigerated	(59, 61)
Cefazolin	10 mg/ml	Heparin 10 and 5,000 units/ml	<20% loss of efficacy at 24 h,	48 h, refrigerated. Stable for 14 days; yellow discoloration	(59, 60)
Ceftazidime	500 mcg/ml	Heparin 100 units/ml	<10% loss of efficacy at 3 days, >30% loss of efficacy at 7 days	7 days	(52, 58)
Ciprofloxacin	0.2 mg/ml	Heparin 10–10,000 units/ml	Compatible for at least 24 h. Data variation across studies	7 days, refrigerated. Stable at 7 days Dwell time up to 72 h between HD sessions	(56)
Daptomycin	25 mg/ml	Heparin 5, 5,000, 10,000 units/ml (reconstituted in LR)	<10% loss in daptomycin concentration at 24 h <10% decrease in daptomycin concentration at 24 h at 37°C *10	At 25°C, 90.7% and 86.7% of daptomycin concentration retained at 48 and 72 h, respectively; 95.2% of gentamicin concentration retained at 96 h	(58, 62)
Gentamycin	1 mg/ml,	Heparin 2,500 units/ml + vancomycin 2.5 mg/ml + cefazolin 5 mg/ml	Compatibility confirmed at 37°C for 72 h	24 h, refrigerated. Dwell time up to 72 h between HD sessions	(58)
	1 mg/ml	Heparin 2,500 units/ml + vancomycin 2.5 mg/ml + cefazolin 5 mg/ml	Compatibility confirmed at 37°C for 72 h	3 days, refrigerated	(58)
Tobramycin	5 mg/ml	Alteplase 1 mg/ml	72 h compatible at 37°C	7 days, refrigerated. Stable up to 48 h	(58)
Vancomycin	25 mcg/ml	Heparin 100 units/ml	At 4°C, vancomycin concentration stable for 14 days; at 37°C, concentration reduced by 15%–37% at 24 h	9 days, refrigerated	(58)
Vancomycin	25 mcg/ml	Heparin 100 units/ml	Compatibility established up to hours 48–72 then discoloration observed	9 days, refrigerated. Stable for 4 days, refrigerated	(58)
Vancomycin	2.5 mg/ml	Heparin 1,000 units/ml	Compatibility established up to hours 48–72 then	9 days, refrigerated. Stable for 4 days, refrigerated	(58)

### Practical considerations of ALT therapies

Biofilms are difficult to eradicate and their successful clearance requires an understanding of their survival strategies and the use of high concentrations of antimicrobials over a sustained duration of time to allow deep penetration of the biofilm matrix (29, 64). The high concentration of antibiotics is necessary for inhibition of bacterial growth and eradication of the biofilm. These solutions could be used not only as adjunct therapy for CRBSI but also for the prevention of biofilm formation. The pre-emptive use of ALT in adult hemodialysis patients has been shown to lead to fewer CRBSI rate and prolonged catheter patency in some randomized controlled studies (40, 65). In their randomized prospective study, Al-Hweish et al. compared infection rates in one group with pre-emptive ALT with Vancomycin-gentamicin-heparin based solution and a second group with routine hemodialysis catheter lock therapy. The incidence of bacteremia and sepsis was significantly lower in the ALT group compared to the routine hemodialysis catheter care group (40). In a similar study, Saxena et al. demonstrated an infection-free and thrombosis-free survival advantage in catheters pre-emptively locked with Cefotaxime-heparin solutions, compared to heparin alone (65). In the largest study to date, comparing the effectiveness of prophylactic antibiotic lock therapy on clinical outcomes in 555 hemodialysis patients, the group whose catheters were locked with a gentamicin/citrate containing solution had significantly less CRBSI rates compared to heparin alone (66). As a result of the heterogeneity of patient population, study quality and design, the most recent Centers for Disease Control (CDC) guidelines do not recommend the routine use of ALT for prevention of CRBSI in long term CVC (67). However, more recent publications provide compelling evidence to the benefit of ALT in the prevention of CRBSI. Moreover, recent large systematic review and meta-analysis of randomized controlled trials have shown ALT to be safe and efficacious in reducing the rates of CRBSI in adult and pediatric patients with CVC (68, 69). These and similar findings are likely to lead to a revision of future guidelines to support routine use of ALT on patients with long term CVCs. In patients with established CRBSI, Khosroshahi et al. demonstrated a significant difference in the success rate of clearing catheter infection in hemodialysis patients with use of 60% ethanol-lock (antiseptic solution) along with systemic antibiotic therapy, and suggested this for routine use (70). A recent meta-analysis of pediatric data showed the addition of ALT to be superior to systemic antibiotics alone for the management of CRBSI and was also associated with less recurrence when compared to patients who were treated with systemic antibiotics alone (35). Although these were mostly oncology patients, the biofilm formation process is likely similar in all patient populations with implantable devices, including CVCs. In the pediatric hemodialysis population, the use of ALT has been shown to be a viable option for reducing systemic antibiotic exposure and antibiotic resistance (71). Although there have been concerns about the emergence of antimicrobial resistance from routine use of ALT in long term CVC, available data suggests the risk to be low (43). Factors that determine the choice of ALT include empiric therapy, culture and sensitivity results, how often the catheter is accessed, stability of the ALT solution, ALT volume variation and inconsistent practices for the documentation of size and length of the catheters, which might lead to subtherapeutic or supratherapeutic dosing. Adverse events associated with the use of ALT are uncommon and are largely related to the individual antimicrobial components of the solutions and could be avoided if solutions are adequately aspirated when accessing the catheters. Although clear guidelines on the role of ALT in the prevention and treatment of CRBSI are currently lacking, available evidence is promising, and future well designed multicenter studies might provide more evidence on the clinical utility of ALT. A list of antibiotic lock formulations and their antibiotic-biofilm interactions can be found in Table 2.

**Table 2:** Antibiotic lock solution-biofilm interaction.

Antibiotic lock solution	Antimicrobial coverage	Biofilm penetration	Duration of use	Reference
Amikacin	P. aeruginosa, S. aureus Gram-neg (ciprofloxacin-resistant)	Slow <25% S. Epidermidis 79%–98%	12 h a day; change every 12 h	(56, 57)
Amphotericin B Liposome- Heparin	Fungi, parapsilosis	Rapid, 80%–100%	Every 24 h	(59, 61)
Ampicillin- Heparin	K. pneumoniae (beta-lactamase positive) "K. pneumoniae (beta-lactamase negative)"	Slow, 80%–100%	Every 8 h	(37, 58)
Capsofungin	C. parapsilosis 1, Fungi	48-h lock @ 2 ug/ml reduced biofilm by 47%	24 h a day for 9 days	(45, 52, 59)
Cefazolin-Heparin	Coagulase-negative staphylococci (CoNS), Klebsiella species, Proteus mirabilis (EKP)	60%–80% Slow penetration	Dwell time up to 72 h between HD Sessions; duration up to 2 weeks	(45, 60)
Ceftazidime- Heparin	K. pneumoniea, E. coli,	68%–70%	8–12 h a day; 7–14 days *9	(45, 58)
Ciprofloxacin- Heparin	M. Morganii 1, Gram neg 2, P. aeruginosa, staph aureus	Rapid & 25%–100% for Pseudomonas aeruginosa 86%–100% for staph aureus and staph epidermidis	12 h a day change every 24 h	(58)
Daptomycin- Heparin	Coagulase-negative staphylococci (CoNS), S. Aureus	Rapid & 100% for S. epidermidis	Dwell times 12–24 h; duration up to 15 days	(45, 61, 63)
Gentamycin- Heparin	Coagulase-negative staphylococci (CoNS), Psuedomonas Putida S. Aureus, Amikacin-Resistant gram neg	Slow & <25% for P. aeruginosa	24 h a day for 9 days	(45, 58, 61)
Tobramycin- Alteplase	P. aeruginosa	Slow & 40% for P. aeruginosa	Sewll times of 72 h with HD sessions	(58)
Vancomycin- Heparin	Coagulase-negative staphylococci (CoNS), S. Aureus, Enteroccocus Hirae, S. epidermidis	Slow Penetration <70% for S. aureus and S. epidermidis “adequate” and slow for S. epidermidis and S. aureus	Vary in neonates. 24 h a day, for 6 days, then 12 h a day for 8 days, total 14 days	(45, 58, 61)

### Author recommendations

In patients with confirmed CRBSI, antibiotic lock therapy should be initiated concomitantly with appropriate systemic antibiotic therapy. Lock therapies should be individualized based on identified organisms and their susceptibilities. If cultures confirm methicillin-resistant *S. aureus* infection, early catheter removal and replacement is recommended. This is the current practice at our center. Catheter salvage and ALT should be pursued only if central venous access is problematic in the patient. Infections with methicillin-susceptible *S. aureus* can be treated with cefazolin lock therapy and if patients remain febrile and continue to have bacteremia 72 h into therapy, the catheter should be replaced. In fungal blood stream infections, infected catheters should always be removed (37). Antibiotic lock solutions should dwell up to 48 h, depending on frequency of catheter use. The catheter should be filled with the volume of antimicrobial solution that fills the entire length of the catheter lumen and this varies depending on the length/ type of catheter and lumen size. The recommended volume is often provided by the manufacturer of the catheter. While the lock solutions dwell in the catheter lumen, the catheter cannot be used until it is aspirated or flushed. The authors recommend aspiration of the instilled volume and avoid flushing of the solution into the patient, to prevent adverse effects. Treatment duration should follow guidelines for systemic antibiotic duration– 3 weeks for uncomplicated bacteremia and 6 weeks for metastatic infection (34, 37). Given the favorable role of ALT in the treatment of CRBSI, the design of clinical pathways and creation of standardized and/or institution specific roadmaps and protocols would be helpful in promoting a wider usage in routine clinical practice.

## Summary and future directions

The clinical burden of CRBSI in pediatric patients with kidney failure dialyzed through a catheter necessitates the development of strategies for the prevention and eradication of biofilms on the surfaces of hemodialysis catheters is paramount. Biofilm formation involves a complex development process and is a survival strategy for pathologic microorganisms. ALT holds promise as a biofilm preventative and eradication tool for clinicians, and although the role of ALT in the management of CRBSI is yet to be clearly defined, available evidence shows clinical advantage in the treatment of these infections. Furthermore, the ability to salvage catheters and avoid catheter removal is of benefit to patients with limited access. Future research must focus on identifying the most effective solutions for preventing catheter colonization and biofilm formation, predicting biofilm susceptibility, and overcoming biofilm resistance. Standardization of practices will be helpful in the design of studies and the development of ALT protocols for use in the pediatric HD population. Adequately randomized studies comparing ALTs with placebo and/or non-antimicrobial solutions, looking at various outcomes such as number of hospitalizations for CRBSI, loss of productive days, care giver time, quality of life and other patient reported outcomes could help justify need for routine ALT therapies.
